# Learning to predict in-hospital mortality risk in the intensive care unit with attention-based temporal convolution network

**DOI:** 10.1186/s12871-022-01625-5

**Published:** 2022-04-23

**Authors:** Yu-wen Chen, Yu-jie Li, Peng Deng, Zhi-yong Yang, Kun-hua Zhong, Li-ge Zhang, Yang Chen, Hong-yu Zhi, Xiao-yan Hu, Jian-teng Gu, Jiao-lin Ning, Kai-zhi Lu, Ju Zhang, Zheng-yuan Xia, Xiao-lin Qin, Bin Yi

**Affiliations:** 1 Chengdu Institute of Computer Applications, Chinese Academy of Sciences, Chengdu, 610041 China; 2 Chongqing Institute of Green and Intelligent Technology, Chinese Academy of Science, Chongqing, 400714 China; 3 University of Chinese Academy of Sciences, Beijing, 100049 China; 4 Department of Anaesthesiology, Southwest Hospital, The Third Military Medical University (Army Medical University), Chongqing, 400038 China; 5 Department of Anaesthesiology, Li Ka Shing Faculty of Medicine, The University of Hong Kong, Hong Kong, China

**Keywords:** In-hospital mortality risk, ICU, Temporal Convolution Network, Attention Mechanism, Time series, Artificial Intelligence

## Abstract

**Background:**

Dynamic prediction of patient mortality risk in the ICU with time series data is limited due to high dimensionality, uncertainty in sampling intervals, and other issues. A new deep learning method, temporal convolution network (TCN), makes it possible to deal with complex clinical time series data in ICU. We aimed to develop and validate it to predict mortality risk using time series data from MIMIC III dataset.

**Methods:**

A total of 21,139 records of ICU stays were analysed and 17 physiological variables from the MIMIC III dataset were used to predict mortality risk. Then we compared the model performance of the attention-based TCN with that of traditional artificial intelligence (AI) methods.

**Results:**

The area under receiver operating characteristic (AUCROC) and area under precision-recall curve (AUC-PR) of attention-based TCN for predicting the mortality risk 48 h after ICU admission were 0.837 (0.824 -0.850) and 0.454, respectively. The sensitivity and specificity of attention-based TCN were 67.1% and 82.6%, respectively, compared to the traditional AI method, which had a low sensitivity (< 50%).

**Conclusions:**

The attention-based TCN model achieved better performance in the prediction of mortality risk with time series data than traditional AI methods and conventional score-based models. The attention-based TCN mortality risk model has the potential for helping decision-making for critical patients.

**Trial registration:**

Data used for the prediction of mortality risk were extracted from the freely accessible MIMIC III dataset. The project was approved by the Institutional Review Boards of Beth Israel Deaconess Medical Center (Boston, MA) and the Massachusetts Institute of Technology (Cambridge, MA). Requirement for individual patient consent was waived because the project did not impact clinical care and all protected health information was deidentified. The data were accessed via a data use agreement between PhysioNet, a National Institutes of Health–supported data repository (https://www.physionet.org/), and one of us (Yu-wen Chen, Certification Number: 28341490). All methods were carried out in accordance with the institutional guidelines and regulations.

**Supplementary Information:**

The online version contains supplementary material available at 10.1186/s12871-022-01625-5.

## Introduction

The in-hospital mortality of patients in the intensive care unit (ICU) is relatively high, ranging from 6.7% to 44.0% worldwide [1, 2]. With the development of critical care medicine, larger amounts of data help doctors to make decisions; however sometimes this can be counterproductive, overwhelming the doctors. Thus, tools that help doctors make decisions based on large amounts of both monitoring results and clinical data are badly needed.

In the past, score-based models, such as simplified acute physiology score (SAPS II), Acute Physiology and Chronic Health Evaluation II (APACHE II), were commonly used in patient evaluations for prediction of mortality risk [3, 4]. When applied to larger populations, the diagnostic performances of score-based models are relatively poor [1, 2, 5–8]. Recently, methods based on artificial intelligence (AI), including conventional machine learning (ML) methods and deep learning methods, have been applied to help doctors’ decision-making by predicting patients’ mortality risk [9–11]. Compared with statistical score-based models, methods based on AI usually have better model performance, which may be related to the advantages of AI methods such as the ability to deal with complex non-linear relationships between variables and patient outcome.

However, there are some limitations of the research mentioned above. One of the most important problems is that the repeated measured variables such as vital signs to predict the mortality risk are replaced with statistical variables, such as maximum, and minimal. In ICU, the overall trend and coupling of changes between different physiological variables may provide prognostic information, which will also help to elevate the accuracy of prediction model [12]. The ideal tool to help doctors’ decision-making requires optimum use of all the associated routine variables, especially time series data, to realize dynamic prediction. However, due to the complexity of the time series data, studies on dynamic prediction using temporal clinical data are limited.

The challenges of predicting mortality risk in the ICU were summarized by Ikaro et al. [12]: Firs, measurements of time series data from each patient vary; moreover, the time interval is irregular. Second, the chosen measurements and the trends of time series data are coupled with each other. In terms of time series models in deep learning, the Long Short-Term Memory (LSTM) [13] and its derivatives Gated-Recurrent Unit (GRU) [14], have been used to predict the mortality risk of ICU patients, which achieved better area under receiver operating characteristic (AUCROC) and area under precision-recall curve (AUC-PR) than conventional score-based models. However, because data are processed sequentially over time, LSTM and GRU have the shortcomings such as high computing load, time consumption, and hardware requirements, as well as vanishing gradients, which result in difficulties in dealing with big data and applying them to clinical popularization. It is widely accepted that deep learning also has other shortcomings such as low explanation capability and larger computing. While the attention mechanism simulates the data processing of the human brain, it is combined with LSTM or other deep learning methods to improve computational efficiency or interpretability [7, 15, 16]. However, the limitations regarding inefficient, particularly when processing long sequences, still exist due to characteristics of the method itself. A better deep learning method that overcomes the current limitations is urgently needed. Recently, a new deep learning method, the temporal convolution network (TCN), was developed, with the characteristics of parallelism, fixed gradient, and smaller memory of training. Furthermore, Bai et al. [17] reported that the TCN has even better performance than LSTM or GRU. Developing an attention-based TCN model may not only elevate the interpretability and reduce the computation complexity but also extend the clinical use due to its higher efficiency for long sequences. Therefore, we aimed to develop an attention-based TCN model to predict the in-hospital mortality risk 48 h after admission in ICUs with time series data and compare the model performances with conventional ML methods, namely, random forest (RF), logistic regression (LR), decision tree (DT) and support vector machine (SVM).

## Materials and Methods

### Ethics and data extraction

**Fig. 1:**
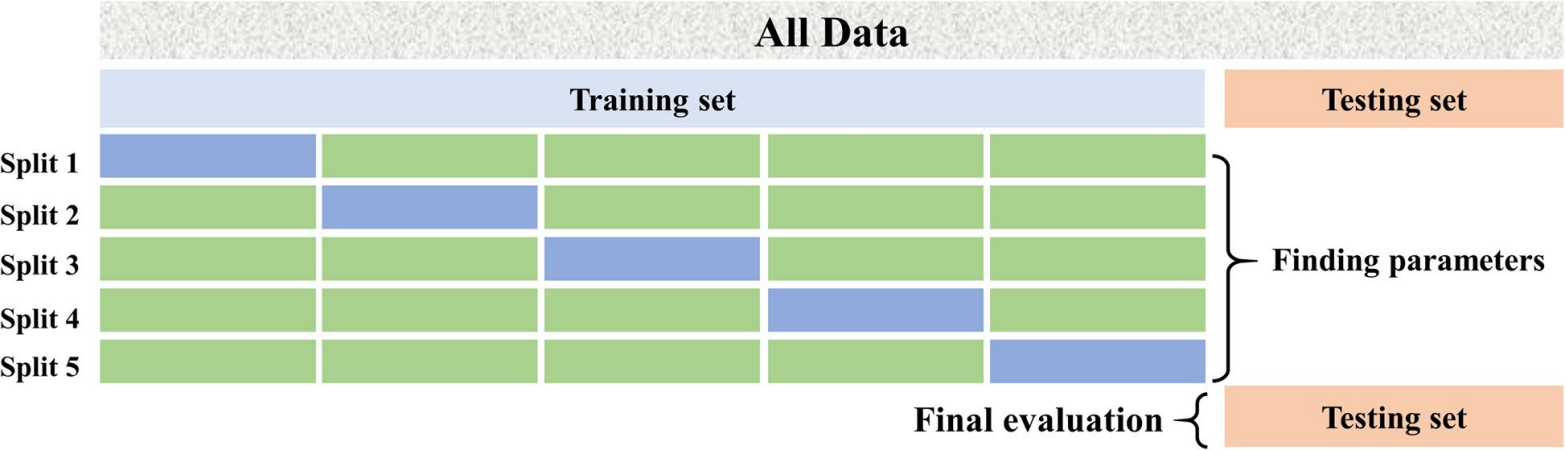
Data partition and verification

Data used for the prediction of mortality risk were extracted from the multi-parameter intelligent monitoring in intensive care (MIMIC) database [18]. The project was approved by the Institutional Review Boards of Beth Israel Deaconess Medical Center (Boston, MA) and the Massachusetts Institute of Technology (Cambridge, MA). The requirement for individual patient consent was waived because the project did not impact clinical care and all protected health information was deidentified [18]. The data were accessed via a data use agreement between PhysioNet, a National Institutes of Health–supported data repository (https://www.physionet.org/), and one of us (Yu-wen Chen, Certification Number: 28341490). All methods were carried out in accordance with the institutional guidelines and regulations. There were 61,532 records of ICU stays in Beth Israel Deaconess Medical Center ICUs, including clinical notes, physiological waveforms, laboratory measurements, and nurse-verified numerical data [18]. The exclusion criteria were as follows: any hospital admission with multiple ICU stays or transfers between different ICUs or wards, which would reduce the ambiguity of outcomes associated with hospital admissions rather than ICU stays; patients younger than 16; patients whose initial ICU stay was missing or less than 48 h; ICU events with no events in the initial 48 h. As a result, a total of 18,094 were included in the final analysis. As shown in Fig. 1, to avoid overfitting, we split the dataset into training set (15331patients, 17,917 ICU stays) and testing set (2763 patients, 3222 ICU stays). Five fold cross validation was performed on the training set to determine the model parameters. We obtained the best model parameters after cross-validation on the training set and obtained the scores of the model on the testing set.

## Data preprocessing

Herein, we mainly focused on common and repeatedly measured variables in ICUs that were effective for reflecting the disease status and efficacy of treatment. We used 17 physiologic variables (shown in Table 1) representing a subset from the Physionet/CinC Challenge 2012 [12]. Up to 17 variables were recorded at least once during the first 48 h after admission. Not all variables were available in all cases. We used all raw values for time series measurements included in the score. For Glasgow Comma Score (GCS), we included GCS verbal response, GCS motor response, GCS eye opening and GCS total as different features. The rest of the variables included weight, height, temperature, respiratory rate (RR), heart rate (HR), diastolic blood pressure (DBP), Mean blood pressure (MBP), systolic blood pressure (SBP), fraction inspired oxygen (FiO_2_), oxygen saturation (OS), pH, glucose, and capillary refill rate (CRR). When the value was more than three standard deviations away from each individual mean value, it was removed. Twelve of the variables were continuous and five discrete. All of the time series variables were re sampled into hourly rate starting from ICU admission. When there was a continuous variable that was missing at a point in time, we filled the data with the nearest neighbour value. When the indicator had no record data during the observation time, we assumed that the nurse did not measure the attribute and that the indicator was considered normal so that we filled the data using the normal value of the attribute. For discrete variables, we performed one-hot encoding. For continuous variables, we performed Z-score normalization to scale the feature values. Each patient’s record was summarized into a visualization data matrix 59 × 48 for 48-h observation period as the input for deep learning.

**Table 1:** Physiological variables to predict the mortality risk of patients in ICU

Sequence number	Physiological variables	Data type
1	Capillary refill rate	Discrete value
2	Diastolic blood pressure	Continuous value
3	Fraction inspired oxygen	Continuous value
4	Glascow coma scale eye opening	Discrete value
5	Glascow coma scale motor response	Discrete value
6	Glascow coma scale total	Discrete value
7	Glascow coma scale verbal response	Discrete value
8	Glucose	Continuous value
9	Heart Rate	Continuous value
10	Height	Continuous value
11	Mean blood pressure	Continuous value
12	Oxygen saturation	Continuous value
13	Respiratory rate	Continuous value
14	Systolic blood pressure	Continuous value
15	Temperature	Continuous value
16	Weight	Continuous value
17	pH	Continuous value

## Model construction for Attention-based TCN

In this work, we developed an attention-based TCN model to predict the mortality risk of ICU patients with time series and static data. The TCN is a convolutional network, which is composed of causal convolution, diluted convolution, and residual connections. The causal convolution makes the TCN a strict temporal model, which uses data from time t and earlier in the previous step to predict the status at time t, when model trained. TCN allows the input of convolution to be sampled at intervals to broaden the field of perception (i.e., to make the most of information) through the use of the dilated convolution. The residual connections enable the network to transmit information across layers, which are usually used to train deep network. In addition, the TCN adds dropout to each hole in the residual module to achieve regularization. An attention mechanism was introduced into the TCN model to elevate the efficiency and the interpretability.

**Fig. 2:**
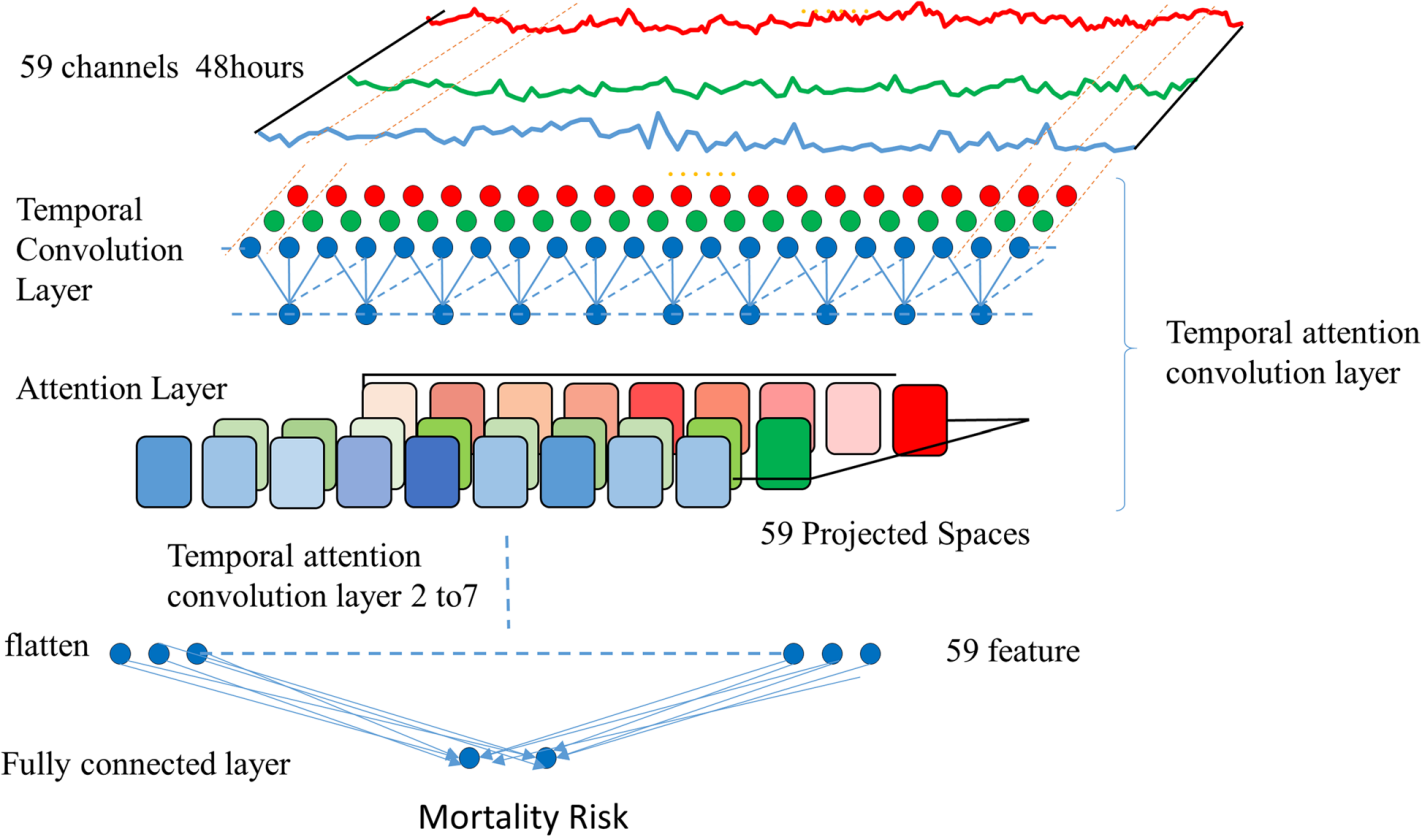
The structure of the attention-based TCN model for prediction of mortality risk in ICU

The structure of the attention-based TCN model was shown in Fig. 2. Patients’ raw data were pre-processed as data flow for model in put; then, the TCN (Temporal Convolutional Network) [17] was directly applied to process the ICU patient's temporal data. The network was similar to the basic structure of the literature [17]. In brief, the model consists of a stack of temporal attention convolutional networks. Each temporal attention convolutional layer was composed of a one-dimensional full convolution layer, self-attention layer and residual layer. Feature extraction was carried out using a one-dimensional causal full convolution layer, and the residual layer was used to deepen the convolution network. The self-attention layer simulates the attention model of human brain and makes the model focus on data relevant to the predicted results. The number of attributes for the patients was 59, so we set the convolution kernel to 3 and the stacked temporal convolutional attention layer to 7. When the network layer was set to 7, the receptive field of the network exactly covered all the patients' input data. The patient's vital signs data are extracted by 7-level TCN and then connected to the attention layer; finally, the mortality risk was predicted by a linear layer. The implementation parameters of the TCN were batch_size = 32, dropout = 0.2, kernel size = 3, levels of TCN = 7, initial learning rate = 0.02, number of hidden units per layer = 59, and optimization algorithm = Adam. The loss function used is binary cross entropy:

$$prob=\frac{1}{1+\mathrm{exp}(-pred)}$$

$$L=-\sum_{i}{label}_{i}*\mathrm{log}\left({prob}_{i}\right)+\left(1-{label}_{i}\right)*\mathrm{log}(1-{prob}_{i})$$

pred: prediction tensor with arbitrary shape.

label: target tensor with values in range [0, 1]. Must have the same size as pred.

## Non-time series model construction

We also predicted the mortality risk by non-time series ML methods such as RF [19], LR, DT and SVM. Due to the limitation of these ML methods, the in-put for these models were not time series data but results of feature extraction (statistical variables, such as the minimum, maximum, average of the variables). Then the preprocessed data were used for model construction and evaluation. For the machine learning models compared in the experiments, the parameters were set through the gridSearchCV method. The corresponding parameters were shown in Table 2.

**Table 2:** The model parameters

Model	The parameter settings
Decision Tree (DT)	criterion = “gini” # The function to measure the quality of a split, supported criteria # are “gini” for the Gini impurity splitter = “best” # The strategy used to choose the split at each node max_depth = None # The maximum depth of the tree min_samples_split = 2 # The minimum number of samples required to split an # internal node min_samples_leaf = 1 # The minimum number of samples required to be at a leaf # node min_weight_fraction_leaf = 0.0 # The minimum weighted fraction of the sum total # of weights required to be at a leaf node max_features = None # The number of features to consider when looking for the # best split random_state = None # It is the seed used by the random number generator max_leaf_nodes = None # Grow trees with max_leaf_nodes in best-first fashion, # if None then unlimited number of leaf nodes class_weight = None # Weights associated with classes, if not given, all classes are # supposed to have weight one presort = False # The data is not presorted
support vector machine (SVM)	kernel = “rbf” # Specifies the kernel type to be used in the algorithm # “rbf” is Gaussian kernel function gamma = “auto” # Kernel coefficient for ‘rbf’ probability = True # Whether to enable probability estimates
logistic regression (LR)	solver = “lbfgs” # The optimized algorithm is “lbfgs” multi_class = “auto” # Determines the multi-class strategy if y contains more than # two classes penalty = “l2” # Specifies the norm used in the penalization, the ‘l2’ penalty is the # standard used in SVC
Random forest (RF)	n_estimators = 100 # The number of trees in the forest

## Model evaluation

Model performance was assessed by overall performance, discrimination, and calibration. The overall performance is determined by the F1 score. The F1 score is defined as the harmonic mean of accuracy and recall, which considers both the precision and the recall equally. Discrimination is the capability to distinguish between those who survival and those who do not 48 h after admission in ICU by AUCROC and the area under the precision-recall curve (AUC-PR). The AUC-PR is sensitive to the imbalanced distribution of the negative and positive data, especially when there is an extreme small portion of positive data. Calibration is assessed by the Brier score via calculating the averaged squared deviation between the predicted probability and the actual outcome.

## Statistical analysis

The statistical analyses were carried out using SPSS software for Windows, V.19.0 (SPSS). Quantitative variables were presented using basic descriptive statistics: mean and SD (for normally distribution data), or median and IQR (for nonnormally distribution data). Comparisons among datasets were performed using the chi-square test, Fisher's exact test, or Kruskal–Wallis test. All statistical tests were two sided, and a *P* value less than 0.05 indicated statistical significance.

## Results

### Data distribution

Ultimately, there were 18,094 patients for analysis. The patient demographics and characteristics of the three datasets are presented in Table 3**.** There were no statistically significant differences in age, sex, and ICU length of stay between the groups. The mortality rate of our cohort was 15.4%. Although the mortality rate of patients in the testing dataset was significantly lower than that of the patients in the training datasets, the mortality rate of patients in test dataset was similar to that of patients in our whole cohort.

**Table 3:** The baseline of patients in training and testing dataset

Variables	Training (*n* = 15,331)	Testing (*n *= 2763)	*P*
Age	67.3(54.0–78.8)	67.7(53.9–79.2)	0.527
Sex (F/M)	6861/8470	1229/1534	0.791
ICU admission			0.014
CCU	2071	380	
CSRU	2768	572*	
MICU	5919	1037	
SICU	2654	455	
TSICU	1919	319	
survival/Death	12,910/2421	2389/374*	0.003
ICU length of stay (hours)	88.8 (63.7–149.9)	86.9 (62.5–147.0)	0.180

Mean (SD) presented for normally distributed continuous variables, while median (IQR) was given to those with non-normally distributed continuous variable. Unless otherwise state n is as indicated in the column headings. The portion of admission in different ICU was statistically compared with the training dataset (*P < 0.05). *F* female, *M* male, *CCU* Coronary Care Unit, *CSRU* Cardiac Surgery Recovery Unit, *MICU* Medical ICU, *SICU* Surgical ICU, *TSICU* Trauma Surgical intense care unit

## Model performance of time series and non-time series models

**Fig. 3:**
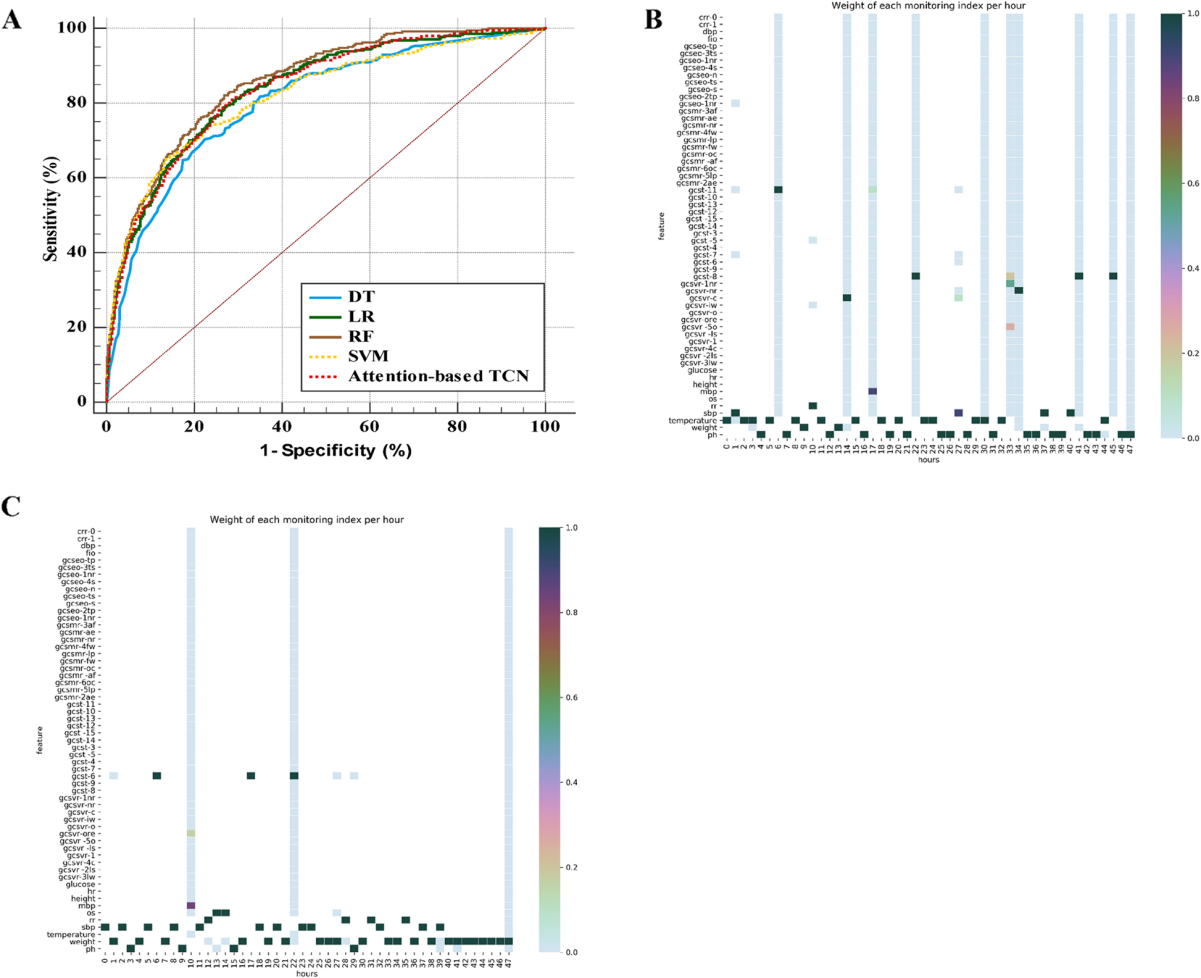
The ROC curves of different AI methods and the typical visualization of attention weight. **A **The ROC curves for predicting ICU patients’ in-hospital mortality 48 h after admission based on different AI methods. **B** The typical heatmap for attention weight of variables and time points for the non-survival patient. **C** The typical heatmap for attention weight of variables and time points for the surviving patient. AI, artificial intelligence; TCN, temporal convolution network; DT, Decision Tree; LR, Logistic Regression; RF, Random Forest; SVM, Support Vector Machine; TCN, temporal convolution network

We evaluated the new model in 3 aspects. First, we compared the attention-based TCN with traditional score-based methods; second, we compared the attention-based TCN with models which do not use time series data; and finally, we compared the attention-based TCN with LSTM that used time series data. The purpose of the comparison with traditional ML models was not to use complex models to compare with simple model but to show that models based on patient time series data are effective in improving the accuracy of predictions compared to models not using time series data. As shown in Tab 4 and Fig. 3 A, compared with the statistical methods, AI methods had larger AUCROC and AUC-PR, which indicated better capacity of discrimination. However, the AUCROC and AUC-PR of the attention-based TCN were smaller than those of the non-time series ML methods, which also had an acceptable discrimination ability. Furthermore, compared with non-time series ML methods, the attention-based TCN had the highest sensitivity (67.1%) and F1 score (0.46). Models with high specificity but lower sensitivity resulting in missing patients who are potentially at risk, which would violate our initial purpose of helping doctors dynamically evaluate the mortality risk of patients. For other time series methods, the sensitivity of the attention-based TCN was much higher than that of model by LSTM (46.1%) based on the same database [7], although there was only a small difference in the AUC-PRs between them. This result indicated that models developed by the attention-based TCN had higher accuracy and a lower omission diagnosis rate than those by LSTM, which may be related to the difference between the input variables. In terms of model calibration, the Brier score of attention-based TCN was higher than that of the other conventional ML models, which may be associated with the high dimension of time series data. Taking the purpose and clinical application into consideration, due to the high sensitivity, F1 score and relative satisfied discrimination ability. Based on these variables, the model performance of the attention-based TCN was the best among the listed methods in Table 4.

**Table 4:** The performances of different ML models for prediction of in-hospital mortality in the test dataset

Methods	Sens	Spec	F1 score	Brier score	AUCROC	AUC-PR
Non-time series methods						
DT	22.7%	96.9%	0.28	0.088	0.804(0.789–0.817)	0.381
LR	35.0%	96.8%	0.43	0.081	0.838(0.824–0.850)	0.459
RF	25.1%	98.5%	0.36	0.077	0.865(0.853–0.877)	0.511
SVM	29.1%	97.9%	0.39	0.080	0.822(0.808–0.835)	0.477
SAPS-II^1^					0.777	0.376
APS-III^1^					0.750	0.357
OASIS^1^					0.760	0.312
Time series methods						
LSTM^2^	46.1%					0.451
Attention-based TCN	67.1%	82.6%	0.46	0.142	0.837(0.824–0.850)	0.454

Statistical quantifications were demonstrated with 95% CI, when applicable. *ML* machine learning, attention-based *TCN* attention-based Temporal Convolution Network, *LR* Logistic Regression, *SVM* Support Vector Machine, *SAPS* Simplified Acute Physiology Score, *APS* Acute Physiology Score, *OASIS* Oxford Acute Severity of Illness Score, ^1^, data referring to Hrayr et al. Scientific Data.2017; ^2^, data referring to Ruo-xi Yu, et al. IEEE J Biomed Health Inform.2019

## Visualization of attention weights at different time points

By visualizing the attention weights, we could clearly see which variables and time points were considered when predicting the risk of death. Typical heatmaps for attention weights of non-survival and survival patients were shown in Fig. 3 B and C. The larger portion of the coloured area in the heatmap of non-surviving patient suggest that the patient is instable. The values of the variables at time points represented by these coloured areas contributed more than other factors to the patient’s death. The time point with most of the coloured variables may correspond to rescue in the clinical reality. In addition to good model performance, the attention-based TCN method also has the potential advantage of better interpretability.

## Discussion

There are several score-based models for predicting the mortality risk, such as SAPS [3], APACHE [20], OASIS [21] and Sequential Organ Failure Assessment (SOFA) [22]. All of these models use non-time series data and are based on statistical methods (i.e., the input data are static data or statistical data, such as comorbidities and the minimum of systolic pressure in the first 24 h), which make it impossible to predict the mortality risk in the first 24 h or to update data for predicting long-term mortality risk. Despite the AUCROCs of the score-based models are satisfied, either the sensitivity or the specificity was poor [23, 24]. It’s not surprising that these models have been modified several times to improve their predictive performance since they initially being published [25]. Recently, for representing the complex, non-linear relationship between clinical variables and the outcome, non-time series AI methods, such as Artificial neural work (ANN), SVM, DT, RF, Naive Bayes, projective adaptive resonance theory (PART) and AutoTriage, were used; demonstrating the ability to predict the mortality risk of patients in ICUs [5, 11, 24, 26, 27] with relatively satisfied model performance. However, in these non-time series methods, all the variables are static or extracted from time series data, which makes it impossible to realize dynamic prediction. The AUCROCs and AUC-PRs of attention-based TCN model were larger than that of conventional score-based models in the same database according to Harutyunyan et al.’s study [8]. It is a pity that Harutyunyan et al. did not show the sensitivity and specificity of conventional models. Regardless of the slight difference in AUCROCs and AUC-PRs among attention-based TCN and other non-time series ML methods, the sensitivity of attention-based TCN was much higher than others. During decision-making in clinical work, doctors should take medical history, physical examination and trend of vital signs into consideration. The ideal model for predicting mortality risk is to take both time series and static clinical data into consideration; moreover, simultaneously realize dynamic prediction. Furthermore, due to the unstable status of ICU patients, sensitivity seems to be more important than specificity, as missing potential patients who are at risk may be fatal for them. In brief, attention-based TCN method was preferable to non-time series methods in predicting the mortality risk of ICU patients. In addition, Hao et al. [28] tried to apply attention-based TCN to language models resulting a significant elevation of model performance, which suggests attention-based TCN is a promising method for sequence modeling.

Recently, Yu et al. [7], Harutyunyan et al. [8] and Song et al. [16] combined two AI methods (including one time series method) to predict the mortality risk of ICU patients with large AUCROCs and AUC-PRs but lower sensitivity (the variables and sensitivity were not presented in Harutyunyan’s study). Along with the low sensitivity, there were other shortcomings in these studies. First, Yu et al.’s and Harutyunyan’s methods were based on LSTM, which addresses time series data sequentially from beginning to end, while TCN can perform parallel processing by causal convolutions in the architecture [17]. Due to the limitations of LSTM, attention-based TCN methods would be more proper for higher dimensions and amounts of data and require less in hardware, which would be more suitable for clinical extension. Second, Yu et al.’s study included vital signs, namely, HR, SBP and temperature, while ours included RR, HR, DBP, MBP, SBP and temperature. Currently, MBP and DBP are widely accepted as important predictors for ICU patients [29–31]. Therefore, it may be insufficient to predict the mortality risk without MBP and DBP. Moreover, some of the variables, such as urinary output in Yu et al.’s study, are the sum or mean of clinical data in a set period time and have a longer acquisition time interval than that of vital signs. Vital signs in our study were more reasonable and easier to obtain than those in Yu et al.’s, while variables more frequently collected could help for dynamic prediction. Third, Harutyunyan et al.’s and Song et al.’s study focused on the algorithms, the clinical value was slightly overlooked. Fourth, these three studies combined an attention mechanism was mainly intended to elevate the efficiency of computing rather than interpretability. Moreover, we furtherly applied the attention-based TCN to predict the patients’ mortality risk 48 h after ICU admission in MIMIC IV (version 1.0) with the same clinical variables and model parameters as that used in MIMIC III. As shown in supplementary Table 1 and 2, the AUC-PR, sensitivity, specificity and F1 score of models based on MIMIC IV were 0.470, 66.0%, 66.0% and 0.35, which were lower than but similar to those based on MIMIC III. Our results suggested that the attention-based TCN had acceptable generalization ability and relatively satisfied robustness. In summary, our attention-based TCN method also had the advantages of higher efficiency, better interpretability and ease of promotion.

**Fig. 4:**
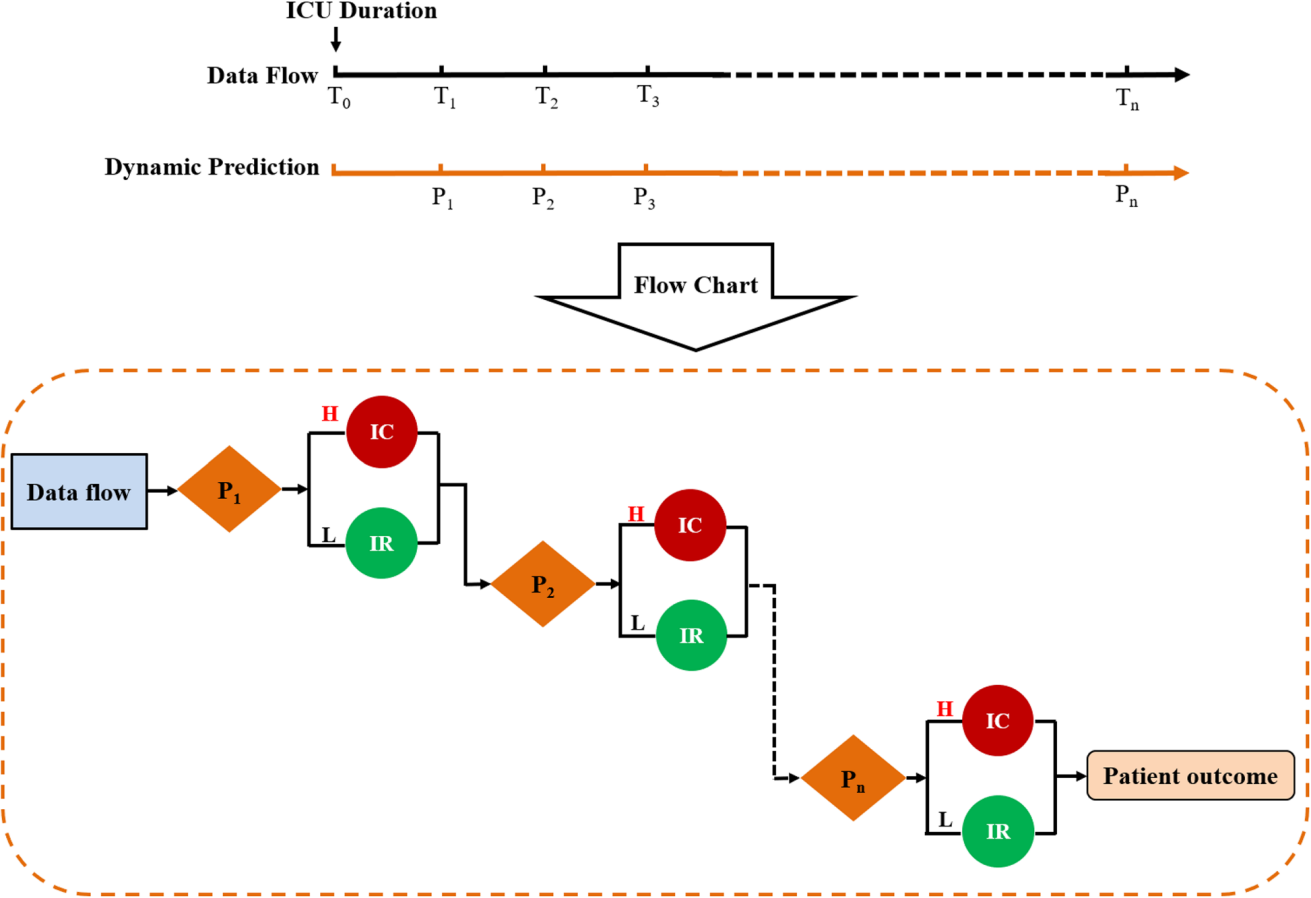
Diagrammatic view of the dynamic prediction of mortality risk in ICU patients by attention-based TCN. (A) Data flow and dynamic prediction are briefly explained by timelines. (B) The instructions of predicting the mortality risk of a new critical patient during the treatment in ICU. T is determined by patient’s main diagnosis and specific condition; P is defined as the prediction of mortality risk at different time point. H, high mortality risk; L, Low mortality risk; IC, Intensive Monitoring and Intensive Treatment; IR, Intensive Monitoring and Routine Treatment

In Fig. 4, we present a diagram for the clinical use of predicting the mortality risk of ICU patients by attention-based TCN methods. For a new critical patient, the patient’s baseline information and monitoring data were put into the attention-based TCN model as data flow after automatically data preprocessing. Then the mortality risk was predicted at different time points according to the patient’s specific condition (here we predict the mortality risk 48 h after ICU admission). If the estimated mortality risk is high, the patient will receive intensive monitoring and intensive treatment; if the estimated mortality risk is low, the patient will receive intensive monitoring and routine treatment. In brief, the whole process is Warning → Intervention → Warning → Intervention → …… → Patient outcome.

There are several limitations in this study. First, although the variables in our study were routine, most of them being time series, some more routine and frequently collected variables would be helpful. New, promising, and repeatedly measured variables should be considered to help elevate the prediction accuracy in further study. Second, clinical data are extracted from one medical center, so the generalization ability of the model and its possibility of clinical application is not validated. Prospective multi-center studies should be carried out to investigate the clinical value of combing TCN with attention mechanism to predict patient’s mortality risk using temporal clinical data.

## Conclusion

Attention-based TCN methods achieved better performance in predicting mortality risk with time series data than non-time series models, suggested that there might be potential for decision-making in ICU by dynamic prediction of mortality risk through continuous data flow.

## Supplementary Information

**Additional file1:** **Supplementary Table 1. **Thedifference in basic information between the training and test datasets in MIMIC III and MIMIC IV. **Supplementary Table 2. **Themodel performance for prediction of in-hospital mortality in the test datasetin MIMIC III and MIMIC IV

## Data Availability

The data that support the findings of this study are available from MIMIC III dataset (https://physionet.org/content/mimiciii/1.4) but restrictions apply to the availability of these data, which were used under license for the current study, and so are not publicly available. Data are however available from the MIMIC III dataset with permission of Massachusetts Institute of Technology Affiliates.

## Abbreviations

AI: Artificial Intelligence
ICU: Intensive Care Unit
DNN: Deep Neural Network
SVM: Support vector machine
GCS: Glasgow Comma Score
ML: Machine learning
DBP: Diastolic Blood Pressure
HR: Heart Rate
TCN: Temporal Convolution Network
APS: Acute Physiology Score
SAPS II: Simplified Acute Physiology Score
CRR: Capillary Refill Rate
LSTM: Long Short-Term Memory
OS: Oxygen Saturation
RNN: Recurrent Neural Network
RF: Random Forest
SBP: Systolic Blood Pressure
RR: Respiratory Rate
MBP: Mean Blood Pressure
LR: Logistic Regression
SOFA: Sequential Organ Failure Assessment
ANN: Artificial neural work
OASIS: Oxford Acute Severity of Illness Score
FiO_2_: Fraction Inspired Oxygen
AUCROC: Area Under the Receiver Operating Characteristic Curve
APACHE II: Acute Physiology and Chronic Health Evaluation II
MIMIC: Multi-parameter Intelligent Monitoring in Intensive Care
