# Supplementary material for: Learning to predict in-hospital mortality risk in the intensive care unit with attention-based temporal convolution network
Source: BMC Anesthesiol. 2022 Apr 23;22:119. doi: 10.1186/s12871-022-01625-5 (PMC9034533; doi:10.1186/s12871-022-01625-5)
Supplement: Supplementary file 1 — Additional file1: Supplementary Table 1. Thedifference in basic information between the training and test datasets in MIMIC III and MIMIC IV. Supplementary Table 2. Themodel performance for prediction of in-hospital mortality in the test datasetin MIMIC III and MIMIC IV [file 12871_2022_1625_MOESM1_ESM.docx]

Learning to Predict in-hospital mortality risk in the intensive care unit with attention-based temporal convolution network

Yu-wen Chen^1,3,4†^, Yu-jie Li^2†^, Peng-Deng^2†^, Zhi-yong Yang^2^, Kun-hua Zhong^1,3,4^, Li-ge Zhang^1,4^, Yang Chen^2^, Hong-yu Zhi^2^, Xiao-yan Hu^2^, Jian-teng Gu^2^, Jiao-lin Ning^2^, Kai-zhi Lu^2^, Ju Zhang^3^, Zheng-yuan Xia^5^, Xiao-lin Qin^1,4*^, Bin Yi^2*^

In order to furtherly prove the generalization of our method, we repeated the analysis on MIMIC IV (version 1.0). The MIMIC IV (version 1.0)[^1^](#_ENREF_1) was released on March 16^th^, 2021, which was new and quite different from MIMIC III. The MIMIC III contains data from 2001-2012, while the MIMIC IV contains data from 2008-2019. The inclusion and exclusion criteria were the same in our manuscript. Finally, in total of 32302 ICU stays were analyzed. After data preprocessing and randomly splitting, 26487 ICU stays were divided into training datasets for model training and cross validation, and the remained 5815 ICU stays were divided into test datasets for model evaluation. The baseline information of the training and test datasets from MIMIC III and MIMIC IV was shown in supplementary Table 1. Finally, we repeated the model construction and evaluation on MIMIC IV data with the same structure and parameter as the one in our manuscript. As shown in supplementary Table 2, the AUC-PR, sensitivity, specificity and F1 score of models based on MIMIC IV were 0.470, 66.0%, 66.0% and 0.35, which were lower than but similar to those based on MIMIC III. Our results showed that the generalization ability of the Attention-based TCN was acceptable.

**Supplementary Table 1. The difference in basic information between the training and test datasets in MIMIC III and MIMIC IV.**

| **Variables** | **MIMIC III** | | ***P*** | **MIMIC IV** | | ***P*** |
| --- | --- | --- | --- | --- | --- | --- |
|  | **Training (n=15331)** | **Test (n=2763)** |  | **Training (n=26487)** | **Test (n=5815)** |  |
| **Age** | 67.3(54.0-78.8) | 67.7(53.9-79.2) | 0.527 | 66.0 (54.0-76.0) | 66.0 (54.0-77.0) | 0.511 |
| **Sex (F/M)** | 6861/8470 | 1229/1534 | 0.791 | 11523/14964 | 2550/3265 | 0.628 |
| **Survival/Death** | 12910/2421 | 2389/374* | 0.003 | 22892/3595 | 5020/795 | 0.842 |
| **ICU stays (h)** | 88.8 (63.7-149.9) | 86.9 (62.5-147.0) | 0.180 | 88.15 (63.4-145.2) | 87.5 (63.4-145.0) | 0.886 |

F, female; M, male; ICU, intensive care unit.

**Supplementary Table 2. The model performance for prediction of in-hospital mortality in the test dataset in MIMIC III and MIMIC IV.**

| **`Datasets** | **Sens** | **Spec** | **F1 score** | **Brier score** | **AUCROC** | **AUC-PR** |
| --- | --- | --- | --- | --- | --- | --- |
| **MIMIC III** | 67.1% | 82.6% | 0.46 | 0.142 | 0.837 | 0.454 |
| **MIMIC IV** | 66.0% | 66.0% | 0.35 | 0.338 | 0.700 | 0.470 |

**Reference**

1. Johnson A, Bulgarelli, L., Pollard, T., Horng, S., Celi, L. A., & Mark, R. MIMIC-IV (version 1.0). PhysioNet2021.
